# Simultaneous production of fatty acids and amino polysaccharides from Norway spruce hydrolysates using oleaginous *Mucor circinelloides*

**DOI:** 10.1038/s41598-025-98549-0

**Published:** 2025-04-23

**Authors:** Cristian Bolaño Losada, Francesca Di Bartolomeo, Alexander Wentzel, Sidsel Markussen, Simona Dzurendova, Boris Zimmermann, Kai Fjaer, Ondrej Slany, Anikó Várnai, Line Degn Hansen, Svein Jarle Horn, Vincent G. H. Eijsink, Vishwanath Patil, Volha Shapaval

**Affiliations:** 1 Faculty of Science and Technology, Norwegian University of Life Sciences, Postbox 5003, 1432 Ås, Norway; 2 Department of Biotechnology and Nanomedicine, SINTEF Industry, 7465 Trondheim, Norway; 3 Faculty of Chemistry, Brno University of Technology, Purkyňova 464/118, 61200 Brno, Czech Republic; 4 Faculty of Chemistry, Biotechnology and Food Science, Norwegian University of Life Sciences (NMBU), Christian Magnus Falsens vei 18, 1433 Ås, Norway; 5 Borregaard AS, 1701 Sarpsborg, Norway

**Keywords:** Lignocellulose, Fermentation, *Mucor circinelloides*, Fatty acids, Amino polysaccharides, Biochemistry, Biotechnology, Microbiology

## Abstract

Lignocellulose is an abundant raw material and renewable carbon source for the production of single cell oils which can replace plant-derived oils in food, feed, fuels, and oleochemicals. *Mucor circinelloides* produces both fatty acids and amino polysaccharides, such as chitin and chitosan. This study evaluates hydrolysates of Norway spruce (*Picea abies*) as a carbon source for their simultaneous production. Cultivation in spruce hydrolysate media yielded 15.8 g/L of biomass, with fatty acids comprising ~ 50% of the cell dry weight and amino polysaccharides up to 8.5%. The fatty acid methyl ester (FAME) content and fatty acid profile were comparable to glucose fermentation. Optimal harvesting times ranged from 72 to 120 h, depending on desired yields. These findings demonstrate that Norway spruce hydrolysates are a viable and sustainable substrate for microbial lipid and polysaccharide production, supporting their potential use in biotechnology and industrial applications.

**Supplementary Information:**

The online version contains supplementary material available at 10.1038/s41598-025-98549-0.

## Introduction

A growing world population, limited fossil resources and environmental concerns regarding their continued use are the main driving forces behind the current quest for alternative, renewable and sustainable sources of lipids and other valuable materials. Oleaginous microbial biomass is a highly attractive alternative source of lipids (single cell oils – SCOs) for a variety of applications in the feed, food, nutraceutical, oleochemical and biofuels industries^1–3^. Compared to plant sources, the production of lipid-rich microbial biomass can be done at fast rates and is independent from variations in seasons and climate. In addition, SCOs and their derivatives can be considered as alternatives to petrochemicals. While some microbial SCOs are very similar to vegetable oils and, thus, suitable for the production of biofuels, other SCOs are similar to highly nutritious and valuable fish oils with a high content of polyunsaturated fatty acids (PUFAs)^4^.

Filamentous fungi are among the less industrially explored SCO producers while exhibiting some of the most desirable features such as rapid growth, a powerful enzymatic apparatus for degrading various feedstocks, high tolerance towards environmental stress, and a capacity to simultaneously produce lipids and other valuable compounds, such as the glucosamine-based polymers chitin and chitosan^5,6^. Due to the valuable physicochemical properties and biological functionalities of glucosamine polysaccharides, there is a growing interest in these biopolymers in the healthcare, chemical and agrobiotechnology industries^7^. Interestingly, while the highly acetylated insoluble form, chitin, is widely found in nature, filamentous fungi are the only known natural producers of the less acetylated soluble forms collectively referred to as chitosan^8^. Although several oleaginous filamentous fungi have been identified and explored for SCO production^4^, relatively high process costs still make the fungal SCOs less attractive compared to conventional lipid sources. The carbon source is one of the major cost drivers in microbial SCO production and is also a major determinant of sustainability^9^. In order to trigger lipid accumulation, carbon needs to be present in high amounts, while access to nitrogen must be limited. Thus, replacing traditional carbon sources by utilizing renewable and abundant non-food related feedstocks, such as lignocellulosic sugars, is of high interest to improve the sustainability of the SCO production process^9^. Moreover, economic viability of fungal bioprocesses can be increased by co-producing valuable products along with SCOs. The fact that *Mucor circinelloides* can perform concomitant production of lipids, chitin, chitosan and carotenoids^3,10–12^ provides an excellent opportunity to increase the sustainability of fungal SCO production.

Coniferous trees are an abundant renewable natural resource. They are relatively easy to cultivate and can produce high-quality pulpwood and lumber. In particular, Norway spruce (*Picea abies*) is among the top most productive silviculture species since it has the capacity to grow on a variety of soil types with profitable biomass accumulation^13^. Low-grade logged trees, including harvested residues and waste, provide an abundant renewable source for microbial biorefineries, and can be valorized into biochemicals and biofuels in a circular bioeconomy. Due to the fact that many microorganisms do not have the required enzymatic apparatus for efficient lignocellulose degradation, the lignocellulosic biomass needs to be mechanically and thermochemically pretreated, followed by enzymatic saccharification of the hemicellulose and cellulose, to yield fermentable sugars^14^. Saccharification and fermentation can either be performed in one reactor in a process called simultaneous saccharification and fermentation (SSF) or in a separate hydrolysis and fermentation setup (SHF)^15,16^.

Sugar-rich hydrolysates originating from spruce have already been applied in several yeast fermentations, to produce ethanol^17–22^ or protein-rich yeast biomass^23,24^. In addition, Norway spruce hydrolysates have been used for the production of biofuels by cyanobacteria^25^, production of docosahexaenoic acid (DHA) and squalene by thraustochytrids^26,27^, as well as production of butyrate, butanediol, polyhydroxyalkanoates, and cellulose by bacteria^28–31^. However, little is known about the potential of using spruce hydrolysates for growing filamentous fungi, although the use of *Mucor indicus* for the production of ethanol from spruce has been reported^32,33^. To the authors’ knowledge, there are no studies reporting production of SCOs or other intracellular metabolites by filamentous fungi growing on spruce hydrolysates, let alone in combination with high-value polysaccharides.

The aim of the present study was, for the first time, to evaluate the suitability of spruce hydrolysates for concomitant production of SCOs and amino polysaccharides (chitin and chitosan) by *M. circinelloides*. We assessed the potential of two different Norway spruce hydrolysates (Excello-90 produced by the Norwegian biorefinery Borregaard AS and an in-house enzymatically produced hydrolysate from BALI^TM^-pretreated spruce obtained from Borregaard AS) as carbon sources in a separate hydrolysis and fermentation (SHF) process setup, using glucose as a control substrate.

## Results and discussion

### *M. circinelloides* growth and substrate consumption

**Fig. 1:**
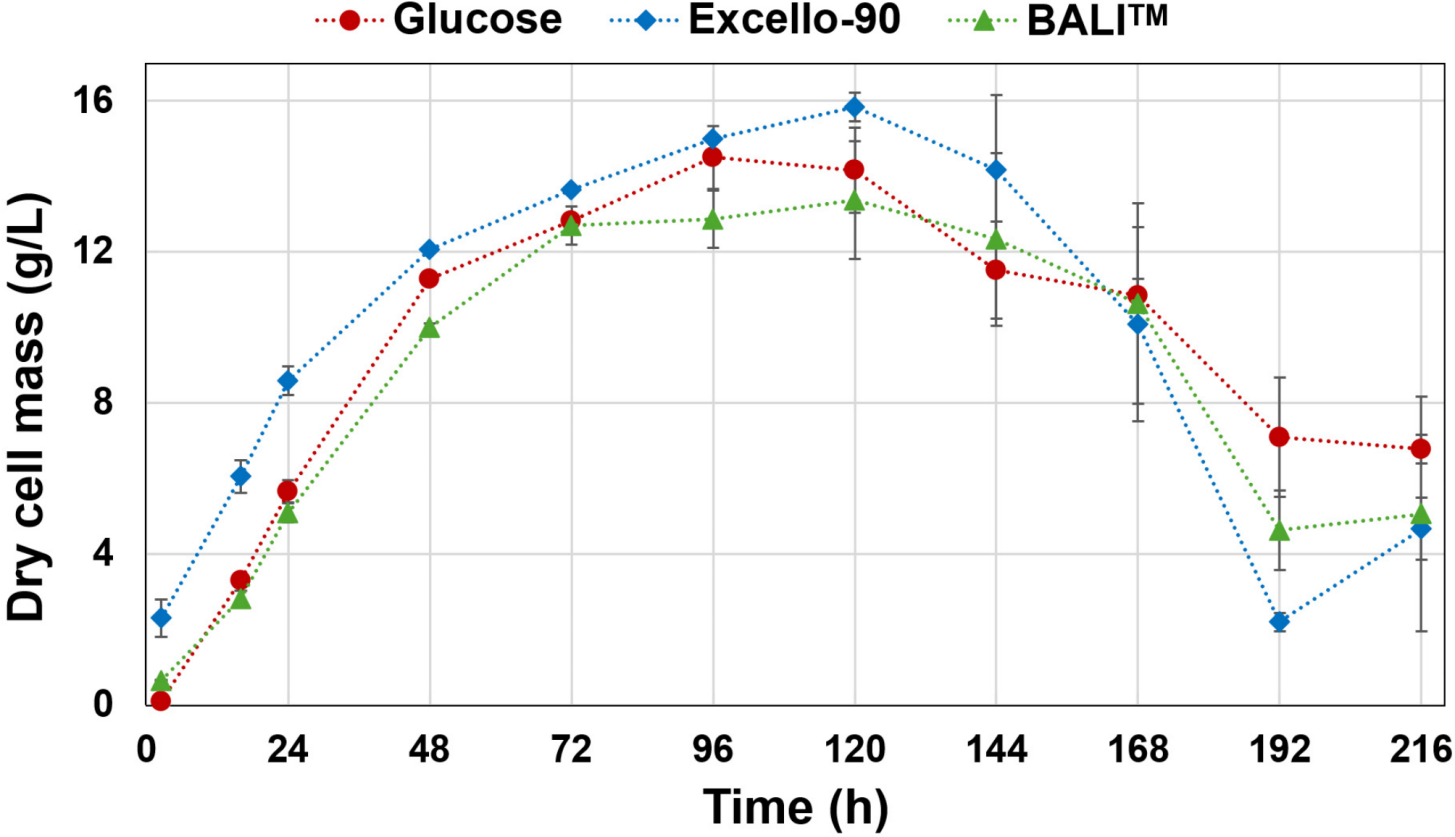
Growth curve of *Mucor circinelloides* cultivated in media with glucose (red), Excello-90 (blue), and the BALI^TM^-pretreated spruce hydrolysate (green). The average values for two biological replicates are displayed with error bars representing the standard deviation.

In this study, two Norway spruce hydrolysates were used as a carbon source for the fermentation of *M. circinelloides* to produce a biomass rich in fatty acids and amino polysaccharides. Figure 1 shows the production of dry cell mass during the fermentation over time.

The growth curves of *M. circinelloides* obtained for the three media reached the different growth phases at nearly the same time points. (Fig. 1). An initial rapid growth phase was observed until 48 h, stationary growth phase was reached at approximately 72 h when, as known from before, nitrogen depletion occurs, followed by an increasingly slower growth phase until 120 h, where the maximum of biomass of each fermentation was reached. Despite equal initial glucose concentrations, minor differences were observed in the values of biomass concentration between treatments at different growth stages.

From 2.5 h to 48 h, the exponential growth phase in Excello-90 was higher and statistically different from the glucose and BALI™ hydrolysate fermentation. This difference could be associated with additional nitrogen present in Excello-90, originated from the enzymes used during the saccharification, which was higher than in BALI™ hydrolysate (Table S1 in Supplementary Materials).

The highest value of biomass was obtained for Excello-90 hydrolysate fermentation, reaching 15.8 g/L after 120 h of cultivation, compared to 13.4 g/L and 15 g/L for BALI^TM^-pretreated spruce hydrolysate and the glucose-based medium, respectively. However, these differences between treatments were not statistically significant, neither at 120 h nor at any time between 72 and 216 h. After 120 h of fermentation, the biomass decreased in all fermentations indicating termination of growth and lipogenesis. From this point, the biomass started to decrease sharply, and the variability between biological replicates increased. This observation suggests the initiation of the death phase in the fungal cells.

**Fig. 2:**
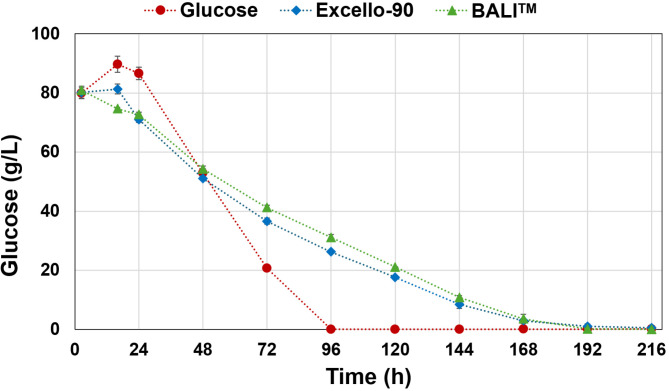
Glucose concentration (g/L) in the culture supernatants during cultivation of *Mucor circinelloides* in media with glucose (red), Excello-90 (blue), and BALI^TM^-pretreated spruce hydrolysate (green). Average values for two biological replicates are displayed, and error bars represent the standard deviation.

Regarding glucose consumption, the glucose-based medium fermentation showed faster glucose consumption than in both hydrolysate-based media fermentations, reaching total depletion after 96 h and coinciding with the maximal dry cell concentration (Fig. 2). While for both hydrolysate fermentations, glucose continued to deplete until 192 h.

**Fig. 3:**
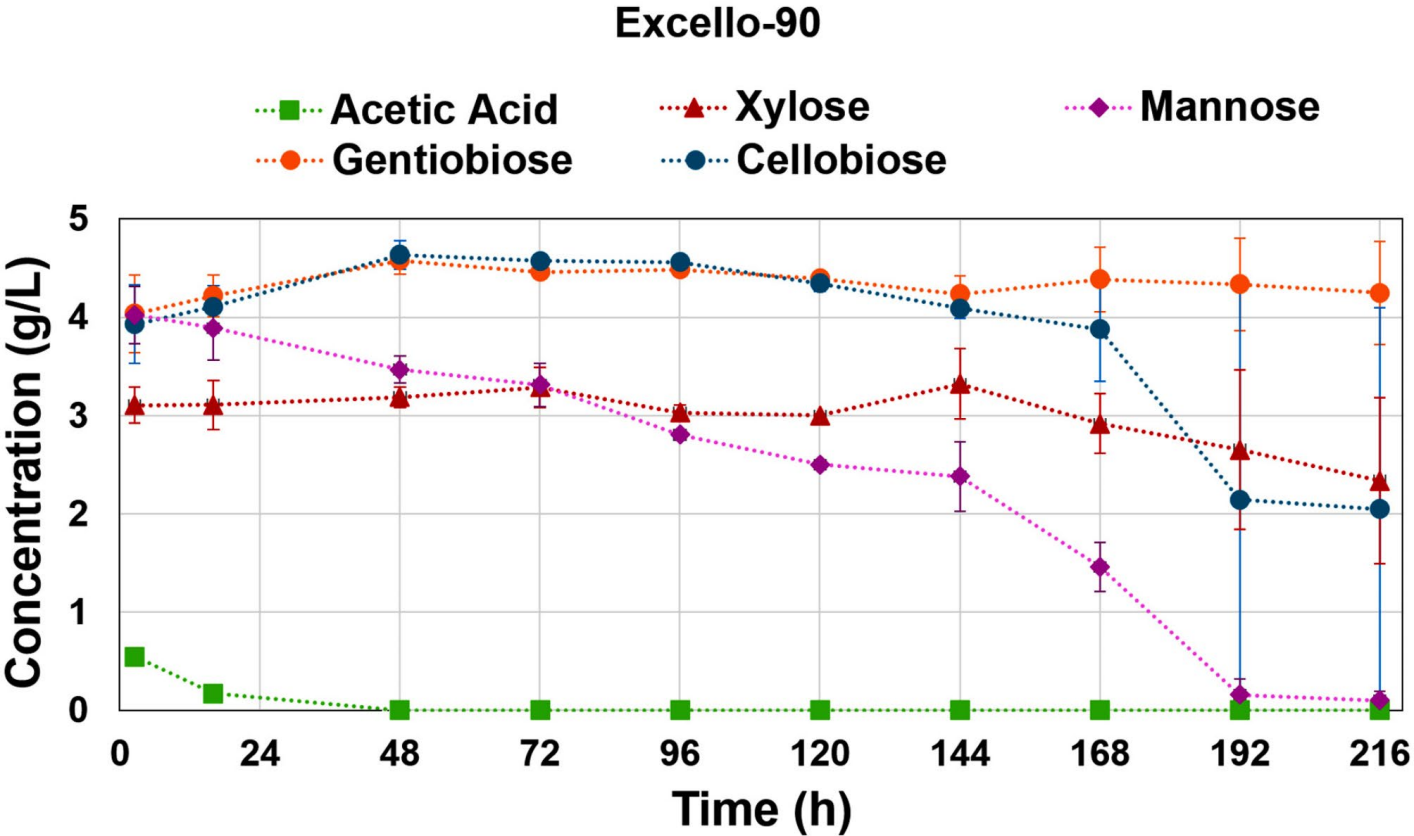

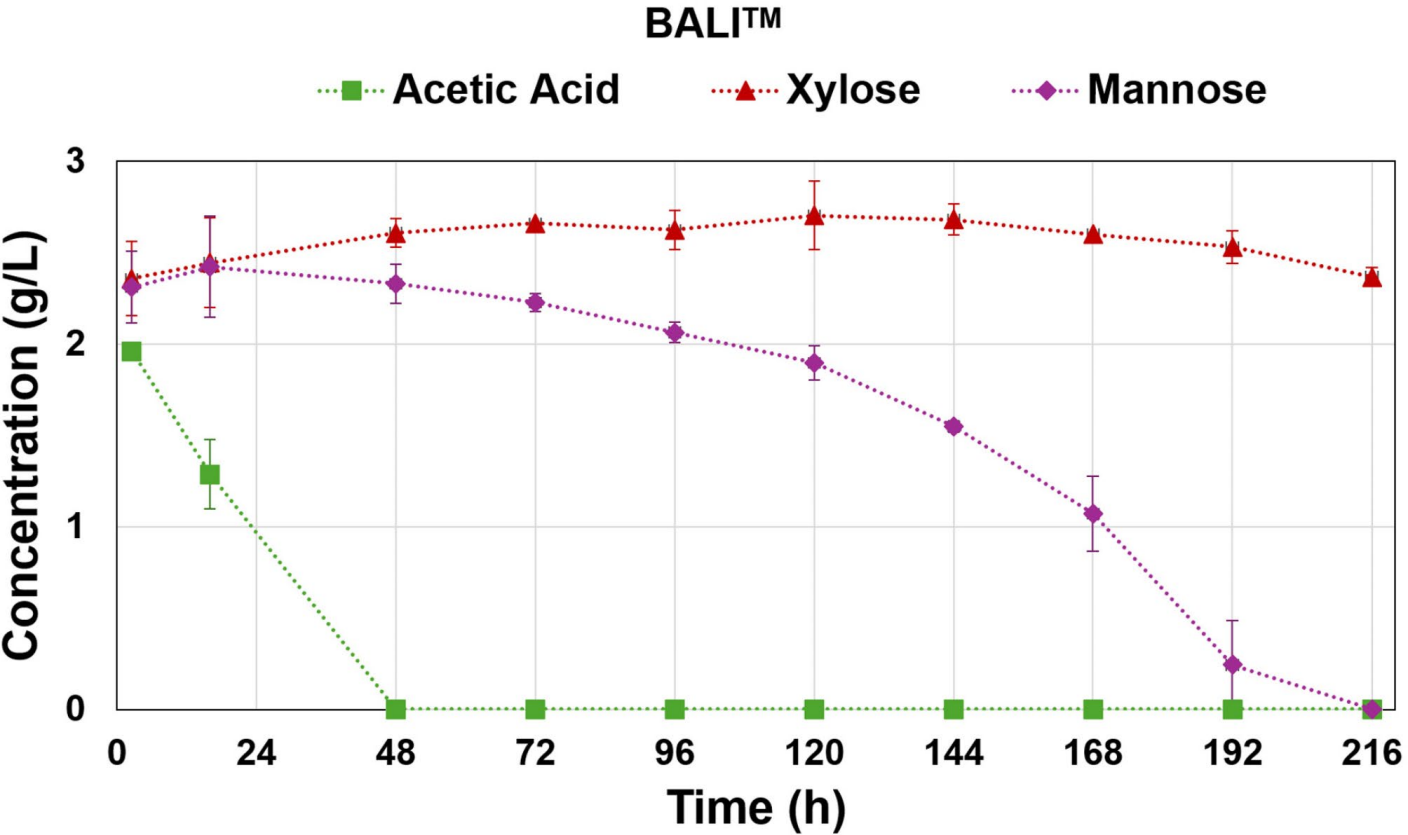
Consumption over time of acetic acid, mannose, xylose, gentiobiose, and cellobiose in (A) Excello-90, and (B) BALI^TM^-pretreated spruce hydrolysate fermentations. The shown values are result of the average of two biological replicates with error bars representing the standard deviation.

The detailed composition of both Excello-90 and BALI™ hydrolysates are summarized in Table S1 in Supplementary Materials. It must be noted that Excello-90 is a product with a high viscosity leading to easy formation of precipitates during storage at 4 °C, this can lead to slight deviations in the measurement of glucose concentration in the hydrolysate product compared to that was observed in the bioreactor mixture. Both hydrolysates contain significant amounts of acetic acid and minor amounts of furfural and HMF, which are known to reduce the activity of the enzymes involved in glycolysis affecting the rate of glucose consumption^34^. The effects of acetic acid and furfural on growth and lipid accumulation were reported in some works using *Mucor sp.*^35^, and more extensively yeast^36^. However, the hydrolysates obtained from the BALI™ process have a very low concentration of furfural, HMF, and lignin-related phenolics. While other processes to obtain sugar hydrolysates from Norway spruce produce furfural and HMF in g/L units^37,38^, the BALI™ process renders concentrations of furfural and HMF in one to two orders of magnitude lower. Therefore, the observed effect on glucose uptake might be mainly due to the presence of acetic acid at a significant concentration, with minor contributions from other inhibitors. Although the fermentation was carried out at pH 6.5 and the pK_a_ of acetic acid is 4.8, a significant portion of it remains in its protonated form. It is known that the protonated form of acetic acid can freely diffuse through the cell membrane producing acidification in the intracellular environment in addition to the previously commented effect on glycolysis^34^. It was observed that acetic acid was consumed from the medium in the first 48 h in both hydrolysate fermentations, which agrees with the previous statement (Fig. 3).

**Fig. 4:**
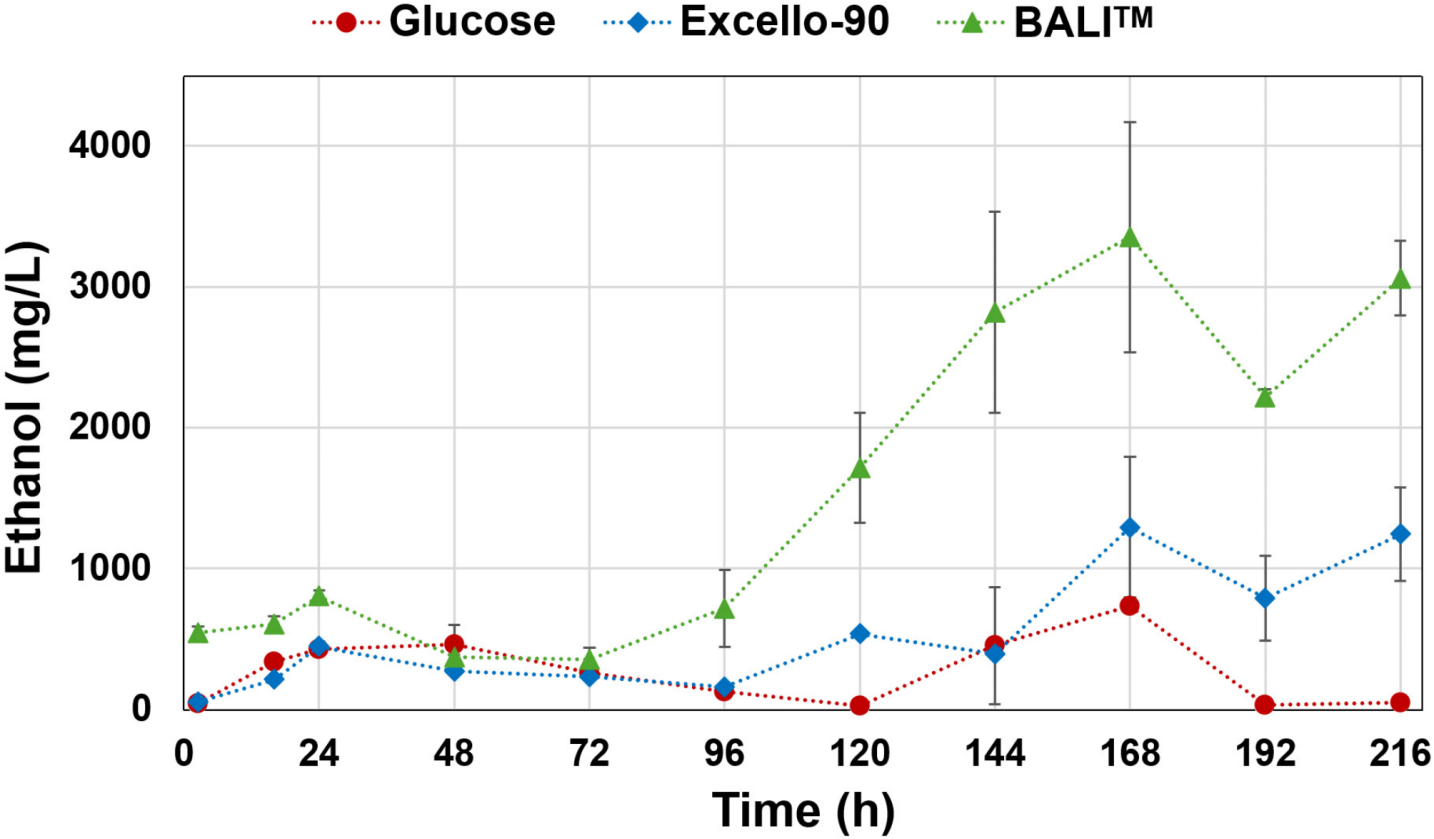
Ethanol concentration (mg/L) in the culture supernatants during cultivation of *Mucor circinelloides* in media with glucose (red), Excello-90 (blue), and BALI^TM^-pretreated spruce hydrolysate (green). Average values for two biological replicates are displayed, and error bars represent the standard deviation.

On the other hand, CO_2_ production continued even after reaching the maximum of biomass concentration (Fig. S1 in Supplementary Materials). The CO_2_ production was higher for both hydrolysates than for glucose fermentations. This was associated with the maintenance of energy consumption to keep the cells active, as well as the production of ethanol, which was shown to increase after 96–120 h of fermentation in all treatments, with higher values in both hydrolysate fermentations. Ethanol production is supported by the fact that *Mucor sp.* is a Crabtree-positive microorganism that can produce ethanol from several sugars under aerobic conditions^39–42^. Interestingly, ethanol production was higher in both hydrolysates than in glucose fermentation, being higher in BALI™ hydrolysate (Fig. 4). This might be related to the higher amount of acetic acid found in BALI™ fermentation. The effect of acetic acid on the production of ethanol was previously reported^35,43^. A possible explanation could be that the intracellular acidification caused by acetic acid, affected pH gradient associated with the electron transport chain in mitochondria. Therefore, it is possible that part of the carbon was diverted to anaerobic respiration to supply energy to the cells and consequently, a higher amount of ethanol was produced. After approx. 170 h of fermentation, a decrease in ethanol content in the media was observed for all three fermentations, likely to cease ethanol production and losses due to evaporation assisted by the aeration and stirring.

In addition to ethanol production, higher CO_2_ production was registered for both hydrolysate fermentations compared to glucose fermentation, that is also related to the additional sugars present in the hydrolysates (Table S1 in Supplementary Materials ). In general, glucose uptake is prioritized over other sugars, which is metabolically regulated by the carbon catabolite repression (CCR) effect^44^. Despite this, for some *Mucor* species it was reported simultaneous uptake of some sugars along with glucose, e.g. lignocellulose hydrolysates, hexoses like mannose can be utilized co-simultaneously with glucose but at a lower rate when they are in similar concentrations^32,35^. While for pentoses like xylose, the CCR effect is stronger and its consumption by *Mucor sp.* has been reported to start only when glucose is depleted^45^.

In both hydrolysate fermentations, mannose started to be consumed after 48 h, increasing the uptake rate as the glucose continued decreasing; meanwhile, xylose was mostly maintained at the same level for the whole fermentation period, and it was only slightly consumed after 168 h, when glucose was near its total depletion (Fig. 3). Additionally, for Excello-90 fermentation, significant amounts of cellobiose and gentiobiose were also present but only cellobiose was shown to be consumed after 168 h (Fig. 3). These oligomers of glucose were not present in the BALI™ hydrolysate (Fig. 3), presumably because the saccharification of pulp was done at much lower solid content than in the case of Excello-90, as evidenced by their considering the final glucose concentrations, 93.4 g/L and 492.4 g/L, respectively. The higher the pulp loading, the higher the glucose concentration produced, which eventually inhibits cellulase activities, such as β-glucosidase, leading to incomplete cellulose processing and accumulation of cellobiose and gentiobiose. Therefore, differences in the presence of these oligomers in the hydrolysates can be explained by the differences in saccharification conditions.

### Production of fatty acids and profile of accumulated fatty acids

**Fig. 5:**
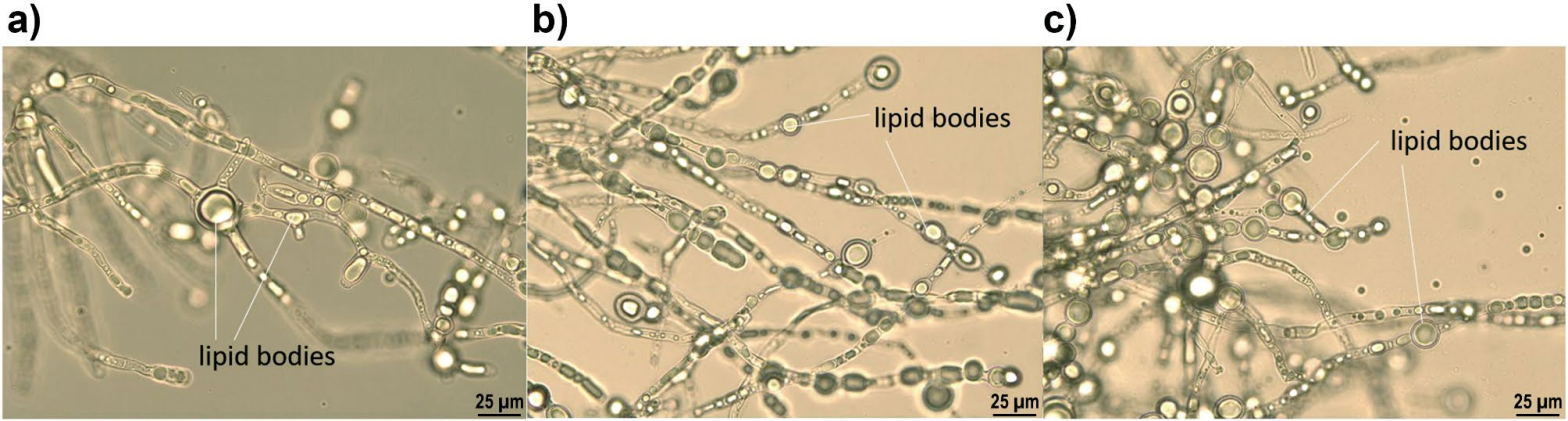
Lipid bodies in fungal cells. The microscopy images show fungal cells from the glucose (A), Excello-90 (B) and BALI^TM^-pretreated spruce hydrolysate (C) -based fermentations after 120 h of growth.

Lipid accumulation in *M. circinelloides* was evident from microscope observations of the fungal hyphae, which contained numerous lipid bodies (Fig. 5). The recorded microscopy images are in accordance with previously reported for *M. circinelloides* containing lipid droplets^4,46^. It is important to note that, while being a dimorphic fungus, *M. circinelloides* exhibited hyphal growth over the whole fermentation period in all fermentation conditions. The macroscopic appearance of the culture was dispersed hyphae without clumps or pellets.

Analysis of fatty acid accumulation was done for biomass sampled at 72 h and later since it is known that the highest rate of lipogenesis occurs at the stationary phase when nitrogen is depleted. As shown in Table 1, in the pure glucose-based medium, a maximum of 52.4% of fatty acids, relative to cell dry weight, was reached already at 72 h of cultivation, which is in good agreement with previously reported levels of lipid accumulation in this fungus when grown on the same medium with glucose^10,47^.

**Table 1:** Total FAME content and fatty acid profiles (w/w%) extracted from *Mucor circinelloides* biomass sampled at 72–216 h of fermentation.

72 h	Glucose	Excello-90	BALI^TM^-pretreated spruce hydrolysate
FAME total	52.4 ± 1.0	45.8 ± 1.0	46.7 ± 0.7
C14:0	1.5 ± 0.1	1.4 ± 0.0	1.6 ± 0.0
C16:0	23.4 ± 0.1	20.9 ± 0.4	20.1 ± 0.3
C16:1	2.4 ± 0.4	2.7 ± 0.2	3.7 ± 0.2
C18:0	8.4 ± 0.1	6.5 ± 0.2	5.8 ± 0.2
C18:1n9c	35.0 ± 0.3	39.6 ± 0.8	40.5 ± 0.5
C18:2n6c	16.5 ± 0.2	16.1 ± 0.2	15.9 ± 0.0
C18:3n6	9.6 ± 0.5	9.7 ± 0.1	9.6 ± 0.3

120 h	Glucose	Excello-90	BALI^TM^-pretreated spruce hydrolysate
FAME total	51.6 ± 2.0	49.8 ± 0.7	50.7 ± 2.0
C14:0	1.4 ± 0.0	1.5 ± 0.0	1.7 ± 0.0
C16:0	21.2 ± 0.1	19.3 ± 0.3	18.6 ± 0.2
C16:1	2.4 ± 0.3	3.0 ± 0.2	3.9 ± 0.0
C18:0	6.9 ± 0.0	5.6 ± 0.1	4.9 ± 0.0
C18:1n9c	36.7 ± 0.2	41.3 ± 0.6	42.3 ± 0.2
C18:2n6c	17.1 ± 0.0	16.5 ± 0.3	16.3 ± 0.1
C18:3n6	11.2 ± 0.3	10.0 ± 0.3	9.8 ± 0.2

168 h	Glucose	Excello-90	BALI^TM^-pretreated spruce hydrolysate
FAME total	49.0 ± 1.0	50.0 ± 1.0	51.9 ± 1.0
C14:0	1.2 ± 0.0	1.6 ± 0.0	1.7 ± 0.0
C16:0	20.0 ± 0.4	18.5 ± 0.1	17.5 ± 0.3
C16:1	2.3 ± 0.3	3.1 ± 0.2	4.0 ± 0.0
C18:0	6.4 ± 0.0	5.3 ± 0.1	4.5 ± 0.0
C18:1n9c	37.1 ± 0.5	42.0 ± 0.4	43.2 ± 0.1
C18:2n6c	17.5 ± 0.1	16.3 ± 0.1	16.4 ± 0.0
C18:3n6	12.3 ± 0.3	10.3 ± 0.3	10.0 ± 0.0

216 h	Glucose	Excello-90	BALI^TM^-pretreated spruce hydrolysate
FAME total	47.00 ± 1.0	51.10 ± 1.0	52.10 ± 1.0
C14:0	1.2 ± 0.0	1.6 ± 0.0	1.7 ± 0.0
C16:0	19.8 ± 0.5	18.2 ± 0.1	17.7 ± 0.4
C16:1	2.3 ± 0.3	2.9 ± 0.1	3.8 ± 0.0
C18:0	6.5 ± 0.2	5.1 ± 0.2	5.7 ± 0.4
C18:1n9c	37.1 ± 1.2	41.6 ± 0.6	41.9 ± 0.4
C18:2n6c	17.6 ± 0.3	16.6 ± 0.2	16.5 ± 0.1
C18:3n6	12.7 ± 0.0	10.6 ± 0.2	10.1 ± 0.2

At the same time, 72 h, both Norway spruce hydrolysate fermentations showed slightly lower values of lipid accumulation, and statistically significant, compared to the glucose fermentation. This observation is aligned with the lower rate of glucose consumption previously explained. Then, the FAME content of both hydrolysate fermentations reached its maximum at 96–120 h, with no statistically significant differences between all the treatments (Table 1).

When the fungal cells possibly reached the death phase, only a decrease in the FAME content was observed for the glucose fermentation, while for both hydrolysates it was relatively stable until 216 h (Table 1). For glucose fermentation, glucose was depleted after 96 h, but CO_2_ continued to be produced (Fig. S1 in Supplementary Materials). Since in the glucose medium, there was no additional sugar to support the maintenance of cell growth, the oxidation of lipids might have been the main source of energy. For the case of both hydrolysates, it was already shown that the energy requirements are fulfilled by consuming additional sugars such as mannose, cellobiose, xylose, and others^48^.

**Fig. 6:**
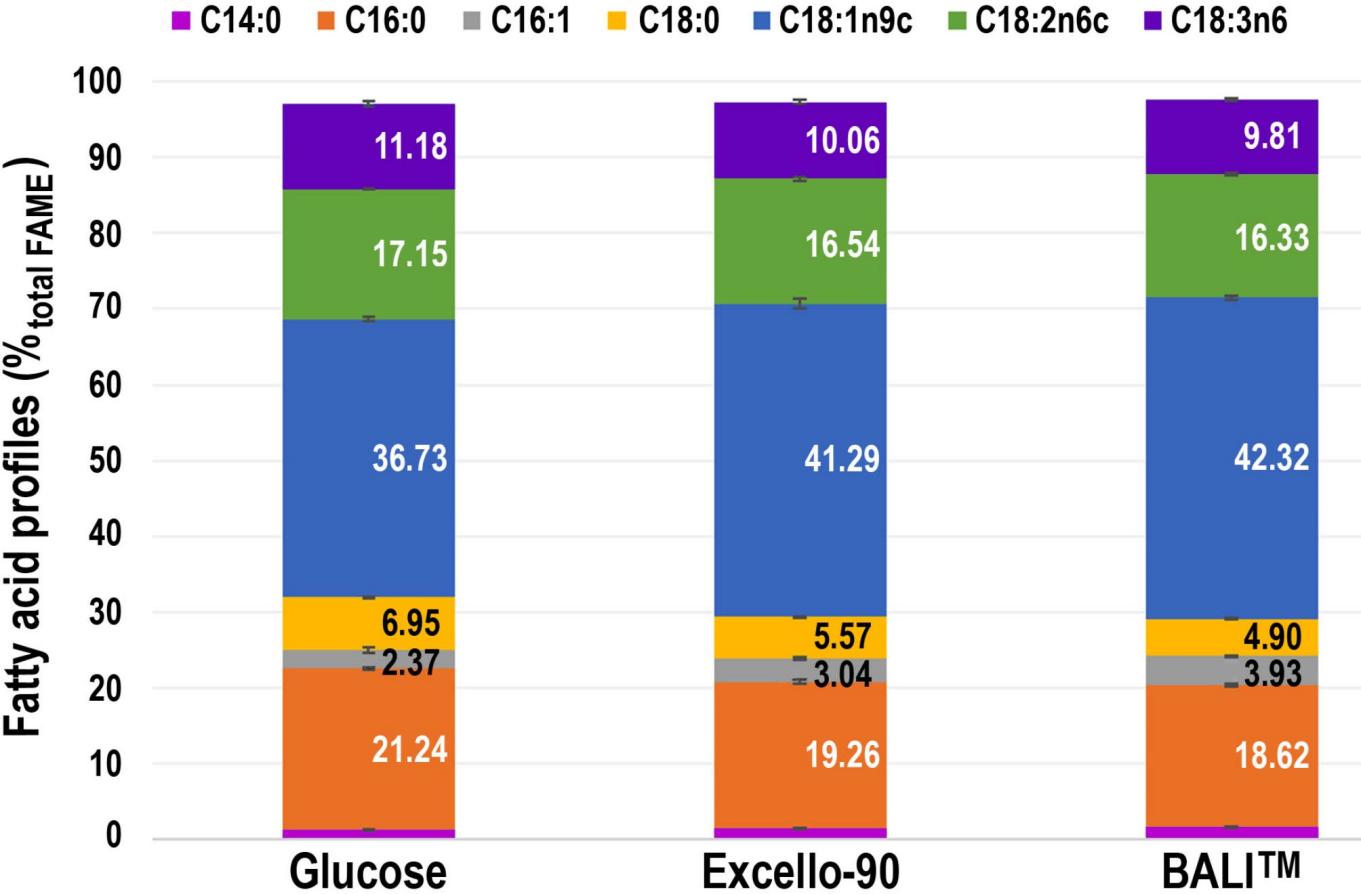
Profiles of fatty acids (%, normalized to total FAME) extracted from *Mucor circinelloides* biomass sampled at 120 h. The plot is based on the average value of the two biological replicates and error bars represent the standard deviation. These data, as well as data for other sampling time points are shown in Table 1.

The profiles of fatty acids produced by *M. circinelloides* were dominated by oleic (C18:1n9c), palmitic (C16:0), linoleic (C18:2n6c), and γ-linolenic (C18:3n6c) fatty acids, regardless of the carbon source (Table 1; Fig. 6) and are in accordance with fatty acid profiles of *M. circinelloides* reported in the literature^4,12,47^. These analyses were done for the time points when both the cell mass and the FAME content were in their maximum (72–120 h), i.e., which are the most relevant from an efficient process point of view.

Oleic acid (C18:1n9c) was the most abundant fatty acid in all fermentations. Its levels were slightly higher in cells grown on both spruce hydrolysates than those grown on pure glucose. Consequently, C18:2n6c and C18:3n6c were in lower percentages, likely due to reduced activity of the Δ12 desaturase, as C18:1n9c serves as their precursor. Lipid profiles of the cells grown in the two spruce hydrolysates (i.e., Excello-90 and the BALI^TM^-pretreated spruce hydrolysate as described in “Production of fatty acids and profile of accumulated fatty acids”) showed nearly identical fatty acid compositions. All in all, the fatty acid profiles of the various cultures were rather similar and in accordance with previously reported^4^. It is worth noting that the small differences that were observed were stable at the most important potential harvesting times (72–120 h). The obtained results also indicate that the two hydrolysates of Norway spruce showed similar performance for total FAME content and fatty acid profile.

### Concomitant production of glucosamine polysaccharides

**Fig. 7:**
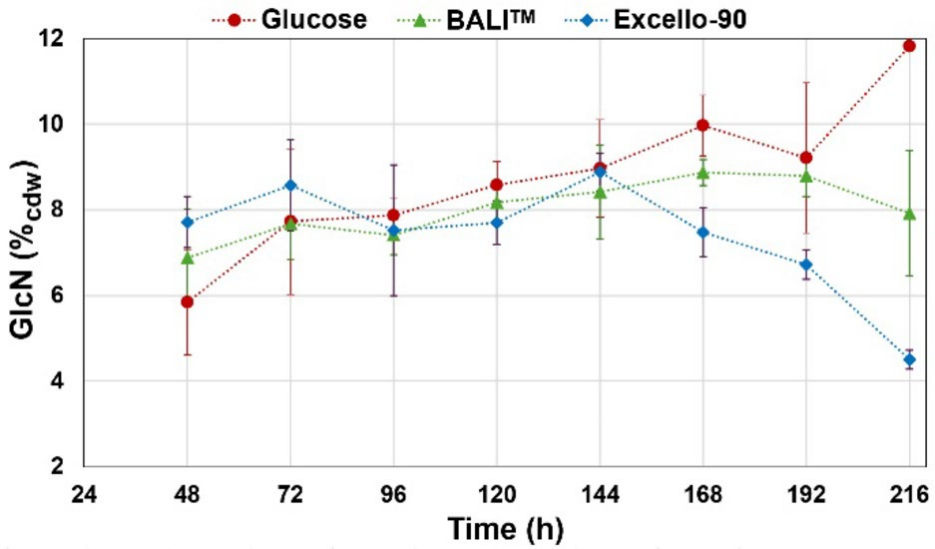
Glucosamine (GlcN) content (% of cell dry weight; cdw) of *Mucor circinelloides* grown on glucose (red), Excello-90 (blue) or the BALI^TM^-pretreated spruce hydrolysate (green). The values represent averages of two biological replicates and error bars show the standard deviation.

Determination of total glucosamine (GlcN) in the *M. circinelloides* biomass was used to estimate the content of chitin and chitosan. The analysis method is based on preparation of an alkali insoluble fraction^49–51^, which means that only polymeric materials are analyzed. These materials are then converted to monomeric GlcN by treatment with strong acid. The GlcN content of *M. circinelloides* grown on the three different substrates at all sampling points is shown in Fig. 7.

The total glucosamine content reached a maximum around 8% (w/w) in all fermentations, remaining stable until 120 h of cultivation. The data showed some minor differences: the GlcN content of the fungal biomass shows a continuous increase during the glucose fermentation (48–168 h), while it stayed relatively unchanged in the fermentations of spruce hydrolysates (48–144 h), however, these differences between treatments were not statistically significant. For the Excello-90 fermentation, the GlcN content went down as the fermentation continued beyond 144 h. While *Mucor circinelloides* demonstrated amino polysaccharide production of up to 8.5% of cell dry weight in Norway spruce hydrolysates, it is essential to evaluate whether this yield is competitive. Previous studies have shown that the content of GlcN in Mucoromycota fungi may reach up to 10% of cell dry weight^52^. However, these previously reported values were derived from studies with lipid-poor cell mass. In the present setup, large amounts of fatty acids were produced, while GlcN production stayed relatively high. Several fungi, such as *Aspergillus*, *Absidia*,* Gongronella*, and *Rhizopus* species, have been studied for chitin and chitosan production, often using agricultural residues or glucose-based media with the reported productivity up to 7.4% of cell dry weight^65^. However, reports on amino polysaccharide production from lignocellulosic hydrolysates are limited. *Mucor circinelloides* shows similar or superior yields compared to other fungi, this highlights its advantage in utilizing lignocellulose-based substrates.

While this study demonstrates the potential of Norway spruce hydrolysates as a carbon source for the simultaneous production of microbial fatty acids and amino polysaccharides, certain limitations should be acknowledged. First, the enzymatic hydrolysis efficiency and compositional variability of the hydrolysates may impact reproducibility and scalability. Additionally, the study focused on a single microbial strain; exploring other oleaginous fungi or microbial consortia could reveal more efficient bioconversion pathways. Future research should also investigate process scale-up, bioreactor configurations, and downstream processing strategies to improve overall productivity and economic feasibility. Finally, extending these findings to other lignocellulosic feedstocks, such as agricultural residues or hardwood species, could broaden the applicability of this approach and support more sustainable biorefinery concepts.

## Conclusions

In this study, we demonstrated the potential of *M. circinelloides* in a separate hydrolysis and fermentation process to convert Norway spruce-derived sugars into microbial biomass rich in fatty acids and amino polysaccharides. The use of spruce hydrolysates resulted in biomass, lipid, and glucosamine yields comparable to those obtained from glucose fermentation, with cell mass concentrations reaching 15.8 g/L, fatty acids comprising ~ 50% of dry weight, and amino polysaccharides ~ 8%. These findings highlight the feasibility of lignocellulosic hydrolysates as a sustainable alternative to conventional carbon sources for microbial oil and biopolymer production. Future research should focus on scaling up fermentation processes, and integrating, both, hydrolysis and fermentation into biorefinery models. Exploring genetic and process engineering strategies could further enhance lipid and amino polysaccharide yields, improving economic viability. This study underscores the industrial potential of Norway spruce hydrolysates for bio-based applications in food, feed, and oleochemical sectors.

## Materials and methods

### Fungal strain and enzymes

The oleaginous *M. circinelloides* VI04473^53^ strain was obtained from the Faculty of Veterinary Medicine of the Norwegian University of Life Sciences (Ås, Norway). Cellic CTec2 was kindly provided by Novozymes A/S (Bagsværd, Denmark). The concentration of protein (81.9 g/L) in Cellic CTec2 was determined using the Bradford method^54^ with bovine serum albumin as standard. Additionally, the filter paper activity (126.5 FPU/mL) was determined according to the IUPAC method^55^.

### Preparation and characterization of the Norwegian Spruce hydrolysates

Norway spruce was used as feedstock to obtain sugar hydrolysates for the fermentation. The feedstock was pretreated by the sulfite-pulped BALI™ process from the Norwegian biorefinery Borregaard (Sarpsborg, Norway)^56^. Two hydrolysates from this material were used in this study: (1) commercial Excello-90 hydrolysate prepared by Borregaard and (2) BALI™ pulp hydrolysate saccharified and produced at NMBU.

For the BALI™ hydrolysate, the pulp (38% DM and around 90% cellulose content)^57,58^ was saccharified in 1.5 L glass bioreactors (Minifors 2, Infors HT, Bottmingen, Switzerland) with 1 L working volume. The reaction mixtures contained 10% (DM w/v) spruce pulp in 50 mM sodium acetate buffer at pH 5.0, and 1mM ascorbic acid (final concentration) was added immediately prior to the addition of Cellic CTec2, dosed at 10 mg protein per g of dry matter. The temperature was maintained at 50 °C and the headspace was left aerobic. A high torque motor (Infors HT, Bottmingen, Switzerland) was used for mixing. The reactors were equipped with two impellers, one pitched blade impeller on the bottom and a marine impeller on top. The stirrer speed was increased stepwise from 100 rpm to 300 rpm during the first 12 h of saccharification. Samples (500 µL) were collected at 4, 24, and 48 h, boiled at 100 °C for 15 min in a heating block (Dry Block Heater 1, IKA, Staufen, Germany), and stored at -20 °C prior to analysis. After 48 h, the hydrolysate was collected by spinning down the remaining solids at 10,000 *g* for 20 min at 4 °C. The supernatants were stored at -20 °C before use.

Acetic acid, cellobiose and gentiobiose were quantified using a Dionex Ultimate 3000 ultra-high performance liquid chromatography (UHPLC) (Dionex, Sunnyvale, CA, USA), equipped with a Rezex ROA-Organic Acid H+ (8%) 300 × 7.8 mm column (Phenomenex, Torrance, CA, USA), operated at 65 °C and coupled to a refractive index (RI) detector 101 (Shodex, Yokohama, Japan). The elution was performed isocratically using 5 mM H_2_SO_4_ at a flow rate of 0.6 mL/min. Glucose, xylose, and mannose were determined by high-performance anion exchange chromatography (HPAEC) using a Dionex ICS 6000 (Dionex, Sunnyvale, CA, USA) equipped with a CarboPac PA1 column kept at 30 °C and a pulsed amperometric detector (PAD). The monosaccharides were eluted isocratically using 1 mM KOH at a flow rate of 0.25 mL/min. Acetic acid, d-glucose, d-xylose, d-mannose, cellobiose, and gentiobiose standards were obtained from Sigma Aldrich (St. Louis, MO, USA). All samples were diluted in distilled water and filtered through a 0.45 μm hydrophilic filter (Millipore, Burlington, MA, USA) before the analysis.

### Experimental design and statistical methods

Six bioreactor fermentations with *M. circinelloides* were performed using two spruce hydrolysates and glucose medium as a control. Each condition was prepared in two independent biological replicates. The fermentation experiments were organized in three steps: (1) cultivation of the fungus on agar plates to prepare a fresh spore suspension, (2) pre-cultivation in nutrient-rich medium in Erlenmeyer shake flasks, and (3) bioreactor cultivation in a nitrogen-limited lipid production medium. An ANOVA test was performed using R 4.4.1 (R Core Team, 2021) to determine significant differences between treatments regarding glucose, biomass, lipid concentration, ethanol, and glucosamine at each time point. The statistical significance was set at *p* < 0.05. The results of the ANOVA test are provided in Supplementary Materials, File 1.

### Preparation of inoculum

To prepare fresh spore suspension, *M. circinelloides* was cultivated on malt extract agar (MEA). MEA plates were prepared by dissolving 50 g of MEA (Merck, Germany) in 1 L of distilled water and autoclaving at 121 °C for 15 min. The agar plate cultivation was performed for 5 days at 25 °C. Fresh spores were collected by spreading sterile 0.9% NaCl solution over the mycelium. The spores were counted using a Burker DHC-B01 C-Chip Disposable Hemocytometer (Nano EnTek Inc., South Korea) and used for the preparation of pre-cultures. The pre-culture medium was composed of 40 g/L glucose, and 10 g/L of yeast extract (Merck, Germany). Each pre-culture was prepared in 500 mL Erlenmeyer flask with 150 mL of pre-culture medium inoculated with 5.10^5^ spores/mL. Each pre-culture was inoculated with spore inoculum prepared from a separate MEA plate. All pre-cultures were cultivated at 25 °C and 150 rpm for 2 days.

### Bioreactor cultivations

Batch cultivations were carried out in 2 L bioreactors (Applikon, Schiedam, Netherlands; working volume: 1.5 L) without baffle plates and with pH, temperature, and dissolved oxygen sensor probes. CO_2_ was continuously measured using an off-gas analyzer (BlueSens GmbH, Herten, Germany). The starting glucose concentration in the pure glucose and the hydrolysates fermentations was 80 g/L. In addition, each medium contained the following nutrients in g/L^59^: yeast extract, 3; KH_2_PO_4_, 7; Na_2_HPO_4_, 2; MgSO_4_·7H_2_O, 1.5; CaCl_2_·2H_2_O, 0.1; FeCl_3_·6H_2_O, 0.008; ZnSO_4_·7H_2_O, 0.001; CoSO_4_·7H_2_O, 0.0001; CuSO_4_·5H_2_O, 0.0001; and MnSO_4_·5H_2_O, 0.0001. The C/N ratio was 100 to ensure early nitrogen depletion triggering lipogenesis as reported in the previous studies^59^. All chemicals were purchased from Merck, USA. Each bioreactor was inoculated with one pre-culture flask of 150 mL (10%, v/v).

All the fermentation experiments were run at 28 °C and pH 6.5, and dissolved oxygen (DO) levels were maintained at 50% by automatic adjustment of the stirrer speed (minimum initial agitation was set to 325 rpm). The aeration rate was constant at 0.5 L sterile air/L culture/min. Dissolved oxygen, agitation speed, pH, and CO_2_ concentration in off-gas were measured and logged on-line using the WinLog software (Sielco Sistemi srl., Italy). Foaming was controlled by adding 1 g/L antifoam A (Sigma-Aldrich, St. Louis, MO, USA. The sampling was done by taking two samples of 10 mL at the following time points: 2.5, 16, 24, 48, 72, 96, 120, 144, 168, 192, and 216 h. All bioreactor cultivations were performed in two biological replicates. The samples were stored at -20 °C until the determination of cell dry weight, concentrations of key growth medium components, and biomass products.

### Preparation of fungal biomass and supernatants for analysis

The cell biomass was separated from the culture supernatant by centrifugation (10 min, 2000 rpm, 4 ^o^C), and washed thoroughly with distilled water using a Millipore vacuum filtration system (Merck MF-Millipore™ Membrane Filter, MCE, 0.45 μm). To determine the cell dry weight, washed biomass from 10 mL of culture was frozen and lyophilized until constant weight in a FreeZone 2.5 freeze-dryer (Labconco, USA) at -50 °C and 0.01 mbar pressure.

For monitoring of glucose and ethanol concentrations, the supernatants were analyzed using a Cedex^®^ Bio Analyzer (Roche, Switzerland) according to the manufacturer’s instructions^61^.

### Determination of glucosamine content

In the first step, alkali insoluble material (AIM) was prepared according to Zamani et al.^62^: 0.5 M NaOH solution (3 mL) was added to 30–50 mg of freeze-dried fungal cells, followed by incubation at 90 °C for 16 h. Subsequently, the sample was centrifuged (5000 rpm, 10 min, 4 °C), and the insoluble material was washed five times with 5 mL distilled water. The supernatant was removed, and the obtained AIM was dried at 70 °C for 48 h. Subsequently, it was mixed with 5 mL of 6 M hydrochloric acid and incubated at 100 °C for 12 h to depolymerize and de-acetylate the chitin/chitosan, and thus convert these polymers to glucosamine (GlcN). Afterwards, samples were processed according to the 3-Methyl-2-benzothiazolinone-hydrazonehydrochloride (MBTH) colorimetric method described by Aidoo et al.^63^ and modified by Slaný et al.^64^. The amount of GlcN was measured colorimetrically using spectrophotometer SPECTROstar Nano (BMG Labtech, Germany) at 650 nm and calculated using a standard curve. These analyses were performed in duplicate for each sample.

### Lipid extraction and gas chromatography analysis

Direct transesterification was performed according to Lewis et al.^65^, with modifications^47^. 2 mL screw-cap polypropylene tube was filled with approximately 20 mg freeze-dried fungal cell mass, approximately 250 mg (710–1180 μm diameter) acid-washed glass beads (Sigma-Aldrich, USA), 1 mg of triacyl glyceride (TAG) internal standard [(100 µL from a 10 mg/mL glyceryl tritridecanoate (C_42_H_80_O_6_, TG(13:0/13:0/13:0); Sigma-Aldrich, USA) in hexane], and 500 µL chloroform. Subsequently, the fungal cell mass was processed in a Precellys Evolution tissue homogenizer (Bertin Instruments, France) at 5500 rpm, by applying a total of six cycles of 20 s. The processed material was transferred to a glass reaction tube by washing the polypropylene tube with 2400 µL of a methanol–chloroform–hydrochloric acid mixture (7.6:1:1, v/v) in three steps (3 × 800 µL). Finally, 500 µL of methanol was added to the glass reaction tube. The reaction mixture was incubated at 90 °C for 90 min, followed by cooling to room temperature, after which 1 mL of distilled water was added. The fatty acid methyl esters (FAMEs) were extracted by the addition of 2 mL hexane followed by 10 s vortex mixing. The reaction tube was centrifuged at 3000 rpm for 5 min at 4 °C, and the upper organic phase was collected in a glass tube. The lower (water phase) was extracted two additional times using 2 mL of a hexane–chloroform mixture (4:1 v/v). The solvent in the pooled organic phases was evaporated under nitrogen at 30 °C and a small amount of anhydrous sodium sulfate was added to the tube. The FAME extract was transferred into GC vials by washing the tube with 1500 µL hexane containing 0.01% butylated hydroxytoluene (BHT; Sigma-Aldrich, USA) followed by 5 s whirl-mixing at slow speed. The lipid extraction and gas chromatography analysis (see below) were performed in duplicate for the sampling points 72, 120, 168, and 216 h of each bioreactor fermentation.

Determination of the total FAME content (expressed as the w/w% of sample dry weight) and fatty acid composition (expressed as w/w% of total FAMEs) were performed by using a 7820 A gas chromatography system (Agilent Technologies, USA), equipped with an Agilent J&W 121–2323 DB-23 column (20 m × 180 μm × 0.20 μm), and a flame ionization detector. Helium was used as a carrier gas. The total run time for one sample was 36 min with the following oven temperature increase: initial temperature 70 °C for 2 min hold, then heating up to 150 °C in 8 min with no hold time, subsequently heating to 230 °C in 16 min with 5 min hold time at 230 °C, then heating to 245 °C in 1 min with 4 min hold time. The injector temperature was 250 °C and 1 µL of a sample was injected (30:1 split ratio, with a split flow of 30 mL/min). For the identification and quantification of fatty acids, the Supelco 37 Component FAME Mix (C4–C24 FAME mixture, Sigma-Aldrich, USA) was used as an external standard, in addition to C13:0 TAG and C15:1 FAME internal standards. Measurements were controlled using the Agilent OpenLAB software (Agilent Technologies, USA).

## Electronic supplementary material

Below is the link to the electronic supplementary material.

Supplementary Material 1

Supplementary Material 2

Supplementary Material 3

Supplementary Material 4

## Data Availability

All data generated or analyzed during this study are included in this published article and its supplementary materials files.
