# Supplementary material for: Simultaneous production of fatty acids and amino polysaccharides from Norway spruce hydrolysates using oleaginous Mucor circinelloides
Source: Sci Rep. 2025 Apr 23;15:14106. doi: 10.1038/s41598-025-98549-0 (PMC12019349; doi:10.1038/s41598-025-98549-0)
Supplement: Supplementary file 1 — Supplementary Material 1 [file 41598_2025_98549_MOESM1_ESM.docx]

**Table S1:** Chemical composition of the Excello-90 spruce hydrolysate (1 L = 1317.156 g) and of an in-house produced hydrolysate of BALI^TM^-pretreated spruce (1 L = 1030 g). NA means the data are not available.

| Component | Excello-90 | BALI^TM^-pretreated spruce hydrolysate |
| --- | --- | --- |
| Glucose | 492.4 g/L | 93.4 g/L |
| Xylose | 51.7 g/L | 5.1 g/L |
| Arabinose | 4.9 g/L | na |
| Galactose | 4.9 g/L | na |
| Mannose | 44.4 g/L | 3.5 g/L |
| Cellobiose | 20.6 g/L | na |
| Gentiobiose | 24.7 g/L | na |
| Fructose | 14.0 g/L | na |
| Glycerol | 12.3 g/L | na |
| Acetic acid | 21 g/L | 2.3 g/L |
| *p*-Coumaric acid | 10.3 µg/g | na |
| Ferulic acid | 35.2 µg/g | na |
| 5-Hydroxymethylfurfural | 23.3 ppm | na |
| Furfural | 24.7 ppm | na |
| Ca^2+^ | 0.183% (w/w) | na |
| Mn^3+^ | 12.8 mg/kg | na |
| Fe^2+^ | 9.04 mg/kg | na |
| Nitrogen | 0.07 g/100 g | 0.01g/100g |
