# Supplementary figures and images for: Simultaneous production of fatty acids and amino polysaccharides from Norway spruce hydrolysates using oleaginous Mucor circinelloides
Source: Sci Rep. 2025 Apr 23;15:14106. doi: 10.1038/s41598-025-98549-0 (PMC12019349; doi:10.1038/s41598-025-98549-0)

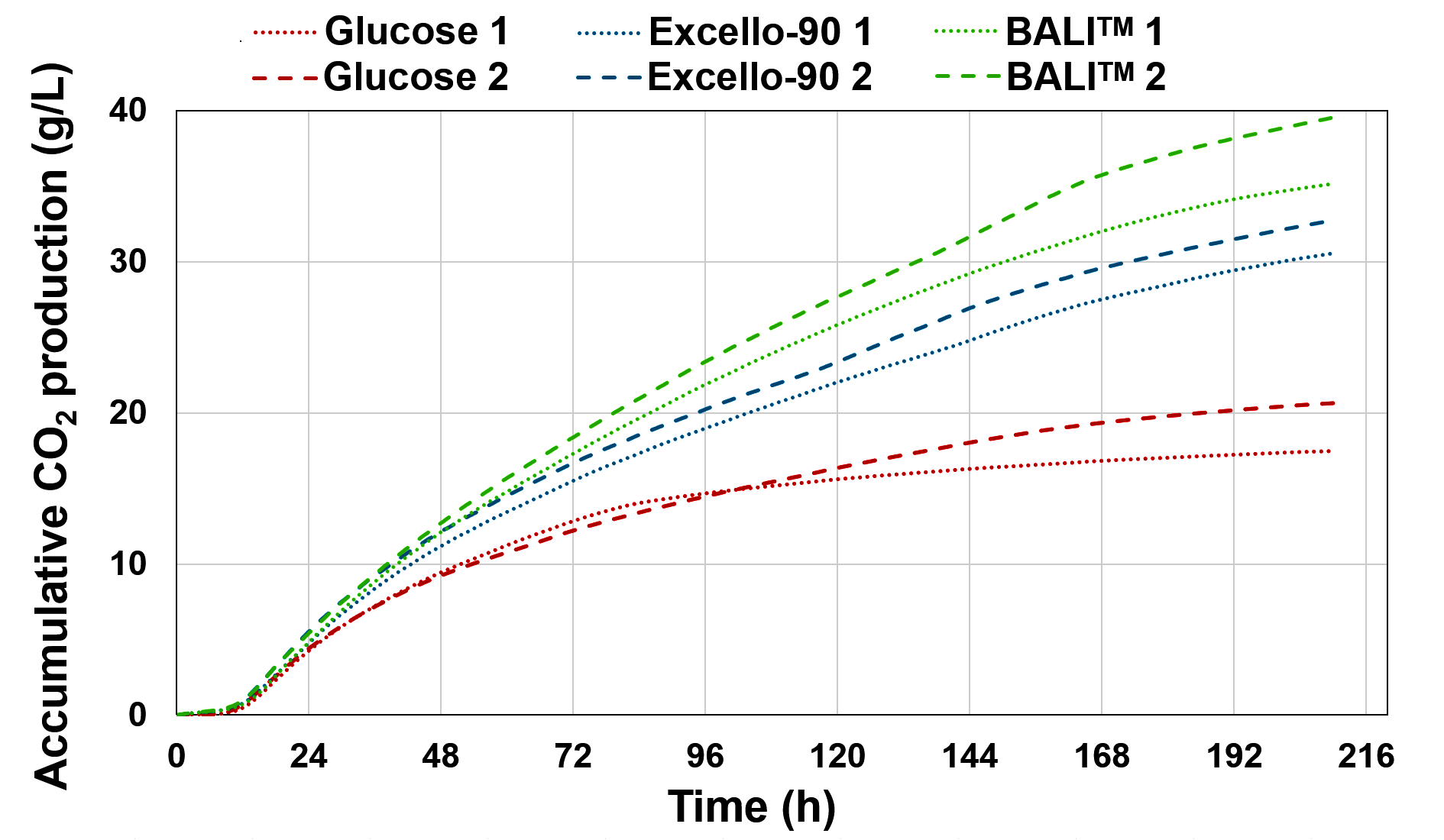

Supplement: Supplementary file 2 — Supplementary Material 2 [file 41598_2025_98549_MOESM2_ESM.tif]
