# Supplementary material for: Simultaneous production of fatty acids and amino polysaccharides from Norway spruce hydrolysates using oleaginous Mucor circinelloides
Source: Sci Rep. 2025 Apr 23;15:14106. doi: 10.1038/s41598-025-98549-0 (PMC12019349; doi:10.1038/s41598-025-98549-0)
Supplement: Supplementary file 4 — Supplementary Material 4 [file 41598_2025_98549_MOESM4_ESM.docx]

**Supplementary Materials**

**Figure S1:** Accumulative CO_2_ concentration in off-gas. All biological replicates are included.

**Table S1 in Supplementary Materials:** Chemical composition of the Excello-90 spruce hydrolysate (1 L = 1317.156 g) and of an in-house produced hydrolysate of BALI^TM^-pretreated spruce (1 L = 1030 g). NA means the data are not available.

**File 1:** ANOVA analysis (diff – difference, lwr – lower bound, upr – upper bound, p adj – adjusted p value, CLD – compact letter display)
